# ﻿*Aletrisguangxiensis* (Nartheciaceae), a new species from Guangxi, China

**DOI:** 10.3897/phytokeys.237.115037

**Published:** 2024-01-19

**Authors:** You Nong, Ke-Dao Lai, Yun-Rui Qin, Gui-Yuan Wei, Ke-Jian Yan, Chuan-Gui Xu, Zi-Yi Zhao, Ren-Chuan Hu, Yun-Feng Huang

**Affiliations:** 1 Guangxi Key Laboratory of Traditional Chinese Medicine Quality Standards, Guangxi Institute of Chinese Medicine & Pharmaceutical Science, No. 20–1 Dongge Road, Nanning, Guangxi, China Guangxi institute of Chinese Medicine & Pharmaceutical science Nanning China; 2 Xishuangbanna Tropical Botanical Garden, Chinese Academy of Sciences, Menglun Town, Mengla County, Yunnan, China Chinese Academy of Sciences Mengla China

**Keywords:** Conservation, flora of China, limestone, Nartheciaceae, taxonomy

## Abstract

*Aletrisguangxiensis* Y. Nong & Y. F. Huang (Nartheciaceae), a new species from Guangxi, China, is described and illustrated. This new species is most similar to *A.scopulorum*, but it can be easily distinguished by its sparsely glandular, 5–18 cm long scape, glandular inflorescence axis, distinctly pedicellate flowers, sparsely glandular, 5–10 mm long pedicel, bract borne at base of pedicel, glabrous perianth divided to the base, strongly recurved or revolute perianth lobes and turbinate, obovoid to oblong-obovoid capsule. An identification key for 24 species and 1 hybrid of *Aletris* is also provided.

## ﻿Introduction

The family Nartheciaceae Fr. ex Bjurzon comprises five genera (Caddick et al. 2002). All species in this family are perennial herbs with short tuberculate or creeping rhizomes, erect stems and terminal spikes or racemes. Various researchers have continuously enhanced its classification (Li and Zhang 2011, Fuse et al. 2012; Tobe et al. 2018). In this family, *Aletris* L. is the largest genus, which contains approximately 21 species distributed in East Asia and North America (Zhao et al. 2012). However, a total of 23 species and 1 hybrid have been accepted according to the Plants of the World Online (POWO 2023).

The genus *Aletris* is characterised by perennial herbs with leaves in basal rosettes, lanceolate to linear blades, racemose to spicate inflorescences, scape simple, erect, usually with a few small, bractlike leaves, flowers bisexual, perianth white, yellow, or golden orange, cylindrical, campanulate or obovoid, with rough abaxial surfaces, six basally connate tepals, six stamens with basifixed anthers, obscurely 3-lobed stigma and fruits capsular, 3-locular, beaked.

During our field surveys in Guangxi in 2020, we found an unusual *Aletris* population that was morphologically similar to the species *A.scopulorum* Dunn. However, this special population is distinctly different from *A.scopulorum*, based on sparsely glandular, 5–18 cm long scape, glandular inflorescence axis, distinctly pedicellate flowers with sparsely glandular, 5–10 mm long pedicel, glabrous perianth divided to the base, strongly recurved or revolute perianth lobes and turbinate, obovoid to oblong-obovoid capsule (Table 1). Therefore, we proposed that this special population may represent a new species. In order to test this hypothesis, we conducted a literature search (Noltie 1994; Yang 1997; Liang and Turland 2000) and examined many specimens of *Aletris* from the Herbaria PE, IBK, GXMI and KUN. Finally, we continued to carry out multiple rounds of field surveys to confirm that this special population represents a new species.

**Table 1.** Main morphological differences amongst *Aletrisguangxiensis*, *A.scopulorum*, *A.gracilis* and *A.cinerascens*.

Morphological traits	*A.guangxiensis*	*A.scopulorum*	*A.gracilis*	*A.cinerascens*
Plant	sparsely glandular	inflorescence axis pubescent	glabrous throughout	glabrous throughout
Leaves	in lax basal rosette, narrowly linear to lanceolate, 4–9 cm × 2–5 mm	in lax basal rosette, linear, 3–15 cm × 2–4.5 mm.	in lax basal rosette, linear, 2–20 cm × 2–7(–9) mm	densely tufted, linear–lanceolate, 4–13 cm × 3–12.5 mm
Scape	5–18 cm	10–35 cm	7–40 cm	8–30 cm
Pedicel	5–8 mm	0.5–3.5 mm	1–10 mm	1–10 mm
Bract and bracteole	bract borne at base of pedicel, bracteole borne on proximal 1/3 of pedicel	bract and bracteole borne on proximal 1/2 of pedicel	bract borne at or near base of pedicel, bracteole borne on proximal 1/2 of pedicel	bract borne at or near base of pedicel, bracteole borne on proximal 1/2 of pedicel
Perianth	White	White	yellowish, whitish or pinkish	yellowish
Perianth lobes	strongly recurved or revolute, linear, 4–7 × 0.2–0.5 mm	erect or slightly recurved, narrowly oblong–lanceolate to linear, 1.5–2.5 × 0.3–0.7 mm	strongly recurved or revolute, narrowly oblong or oblong, 2–3 × 1 mm	strongly recurved or revolute, narrowly lanceolate, 3–4.5 × 1–1.5 mm
Capsule	turbinate, obovoid or oblong–obovoid, distinctly angular, 2–4 × 2–3 mm	subglobose, 3–3.5 × 2.5–3 mm	narrowly ovoid, 4.5–7 × 2.5–3.5 mm	oblong–ovoid or ± ellipsoid, 5–7 × 3–3.5 mm

## ﻿Materials and methods

The new species were described based on field observations that were conducted in March to May and examination of herbarium specimens at GXMI. Other related *Aletris* species were examined based on online images from Kew Herbarium Catalogue (http://apps.kew.org/herbcat/gotoHomePage.do) and JSTOR Global Plants (http://plants.jstor.org/) and Chinese Virtual Herbarium (https://www.cvh.ac.cn/). Morphological characters that distinguish it from all other species in the genus of *Aletris* are used. We also observed living plants of the new species at flowering and fruiting time (March to May). We observed characters of stems, leaves, pedicels, flowers, receptacles, petals, stamens, gynoecium, carpels, size of flowers, size and shape of petals, number of stamens and the shape of gynoecium and fruit.

Descriptions were written from herbarium specimens. Measurements were made with a tape measure and calipers. The structure of the indumentum and its distribution were observed and described under a dissecting microscope at magnifications of more than 20×. Additional information on locality, habitat, ecology, plant form and fruits were collected in the field and taken from herbarium labels. Conservation threat assessment followed IUCN Categories and Criteria (IUCN 2022).

## ﻿Results and discussion

### ﻿Taxonomy

#### ﻿ Aletris guangxiensis

Taxon classificationPlantaeDioscorealesNartheciaceae

Y.Nong & Y.F.Huang sp. nov.

D80EFD82-2EF2-558B-9133-B04AC87DCA9B

urn:lsid:ipni.org:names:77334668-1

Figs 1 , 2 , 3 , 4

##### Diagnosis.

*Aletrisguangxiensis* is most similar to *A.scopulorum*, but it differs by inflorescence axis sparsely glandular (vs. pubescent), pedicel 5–8 mm (vs. 0.5–3.5 mm), bract borne at base of pedicel (vs. bract borne on the proximal 1/2 of the pedicel), lobes strongly recurved or revolute, linear, 4–7 × 0.2–0.5 mm (vs. erect or slightly recurved, narrowly oblong–lanceolate to linear, 1.5–2.5 × 0.3–0.7 mm). At first glance, it also looks similar to *A.gracilis* Rendle and *A.cinerascens* Wang & Tang, but differs by its inflorescence axis sparsely glandular (vs. glabrous), pedicel 5–8 mm (vs. 1–10 mm), perianth white (vs. yellowish, whitish or pinkish/yellowish). More detailed morphological differences amongst the four species are provided in Table 1.

**Figure 1.**
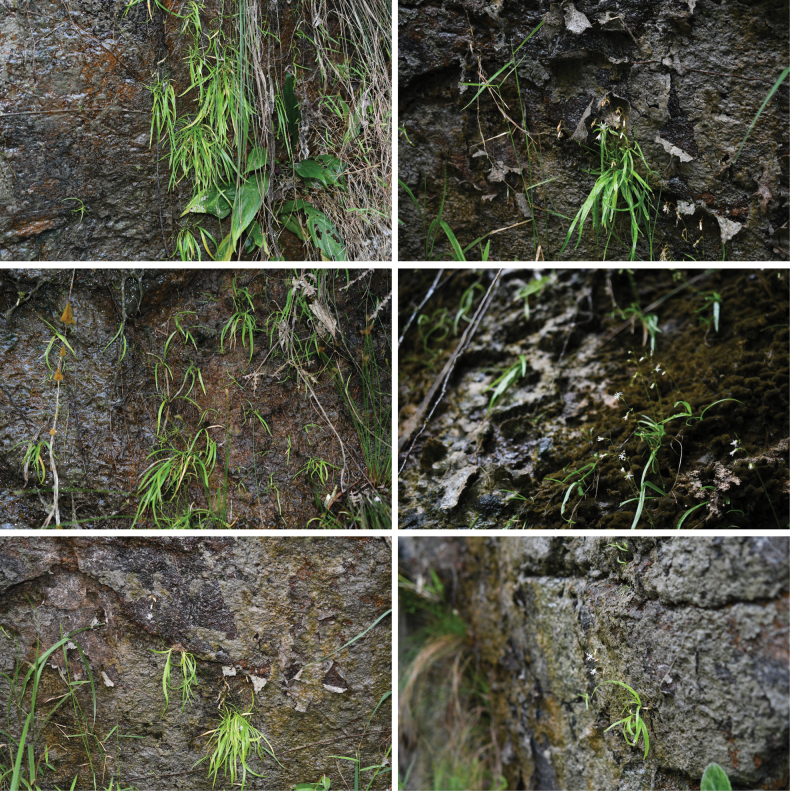
Habitat of *Aletrisguangxiensis* on the moist cliffs next to streams. [Photographed by You Nong and Ke–Jian Yan].

##### Holotype.

China. Guangxi: Cenxi, 22°44'5"N, 110°51'59"E, alt. 320 m, on the cliff next to the stream, 23 April 2020 *Y Nong NY2020042301* (holotype GXMI! isotypes IBK!).

**Figure 2.**
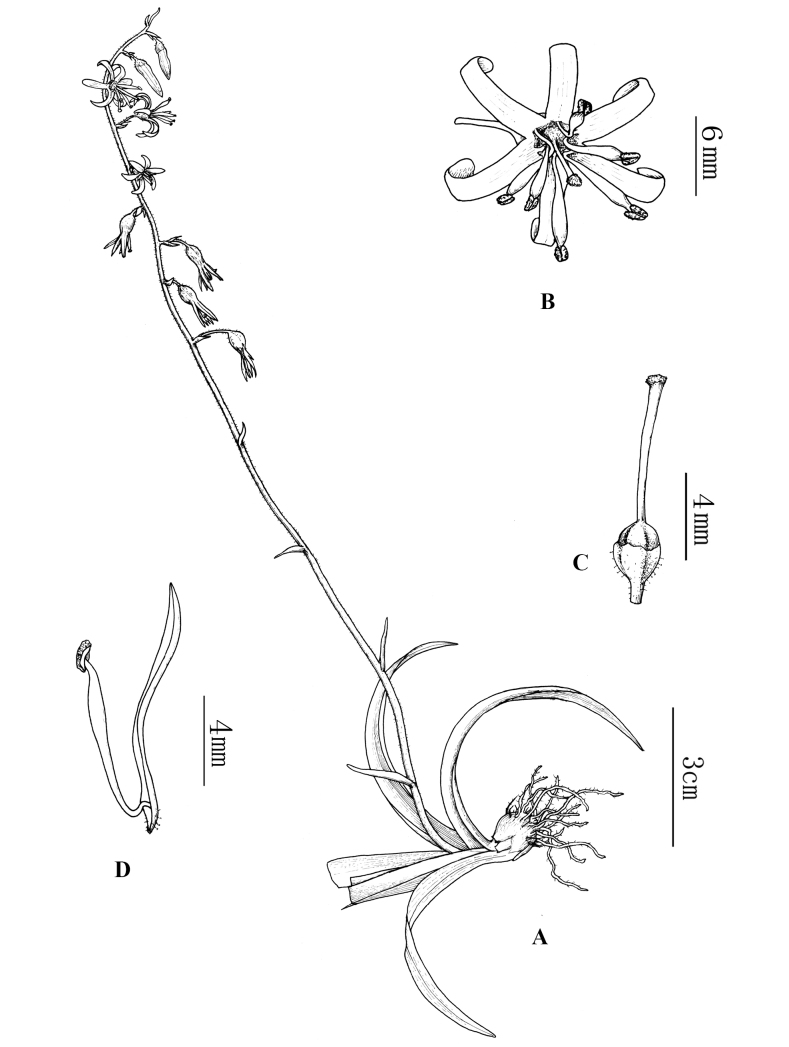
Line drawing of *Aletrisguangxiensis***A** flowering branch **B** flowers **C** Ovary and stigma **D** Filaments of stamens and perianth [Drawn by Xin–cheng Qu from *Y Nong NY2020042301* (GXMI)].

##### Description.

Herbs. Roots usually fibrous. Leaves in basal rosette, narrowly linear to lanceolate, 4–9 cm × 2–5 mm. Scape 5–18 cm, sparsely glandular, bract–like leaves 3–10 mm long in the middle and lower part. Raceme 2.5–9 cm, laxly 2–10(or more)–flowered; axis glandular. Flowers distinctly pedicellate; pedicel 5–8 mm, sparsely glandular, subtended by a bract borne at base of pedicel and bracteole borne on proximal 1/3 of pedicel above bract; bract and bracteole lanceolate, 2–4 mm, shorter than flower, apex subacute. Perianth white, glabrous, divided to the base; lobes strongly recurved or revolute, linear, 4–7 × 0.2–0.5 mm, apex obtuse. Filaments of stamens adnate to perianth, 3–4 mm. Style 0.2–0.5 mm; stigma conspicuously thickened, capitate. Fruits capsular, 3–locular; capsule turbinate, obovoid or oblong–obovoid, distinctly angular, 2–4 × 2–3 mm.

**Figure 3.**
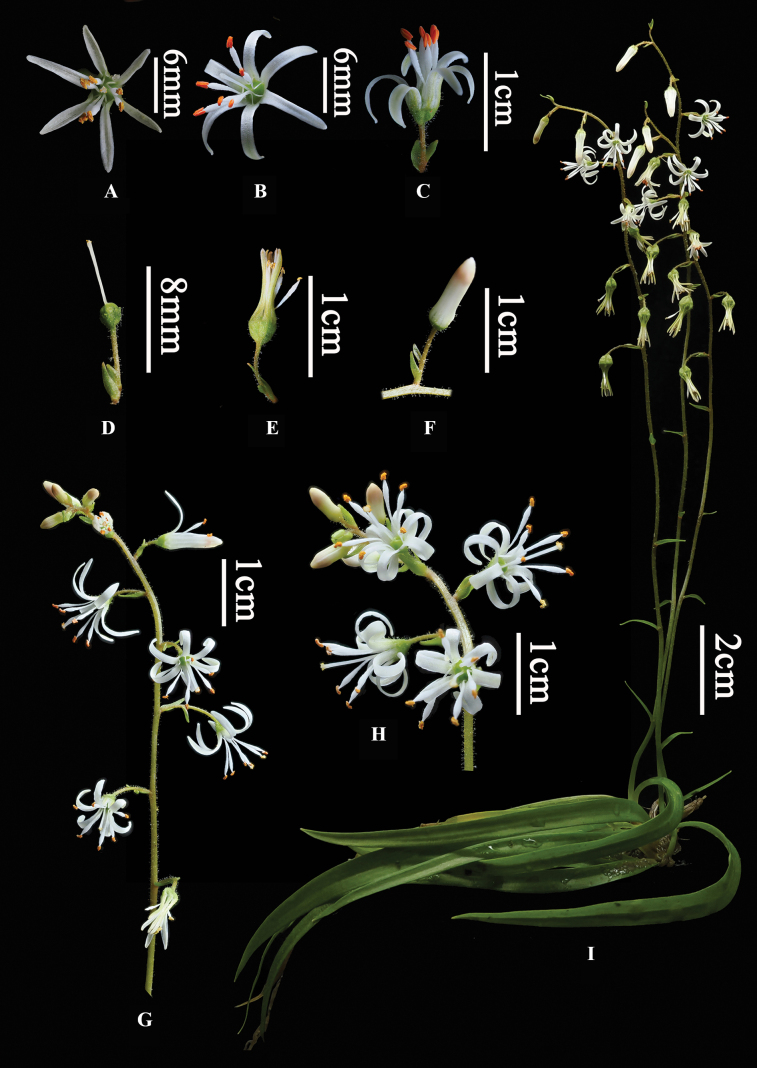
*Aletrisguangxiensis***A** flower (front view) **B, C** flower (lateral view) **D** ovary and stigma **E** young fruit **F** inflorescence node with flower-subtending bract and flower bud, pedicel with bracteole in its proximal part **G** inflorescence **H** flowers **I** plant [Photographed by Ke–Jian Yan from *G.Y. Wei WGY2023033001* (GXMI), edited by Yuan Fang].

##### Phenology.

Flowering and fruiting in March to April.

##### Etymology.

Guangxi is located in the southwest of China and is a biodiversity hotspot where many new species or new species records have been found (Hu et al. 2019; Luo et al. 2020; Feng et al. 2021; Xin et al. 2021; Huang et al. 2022; Nong et al. 2023). The new species, *A.guangxiensis*, is found in this region and is named after the geographic location.

**Figure 4.**
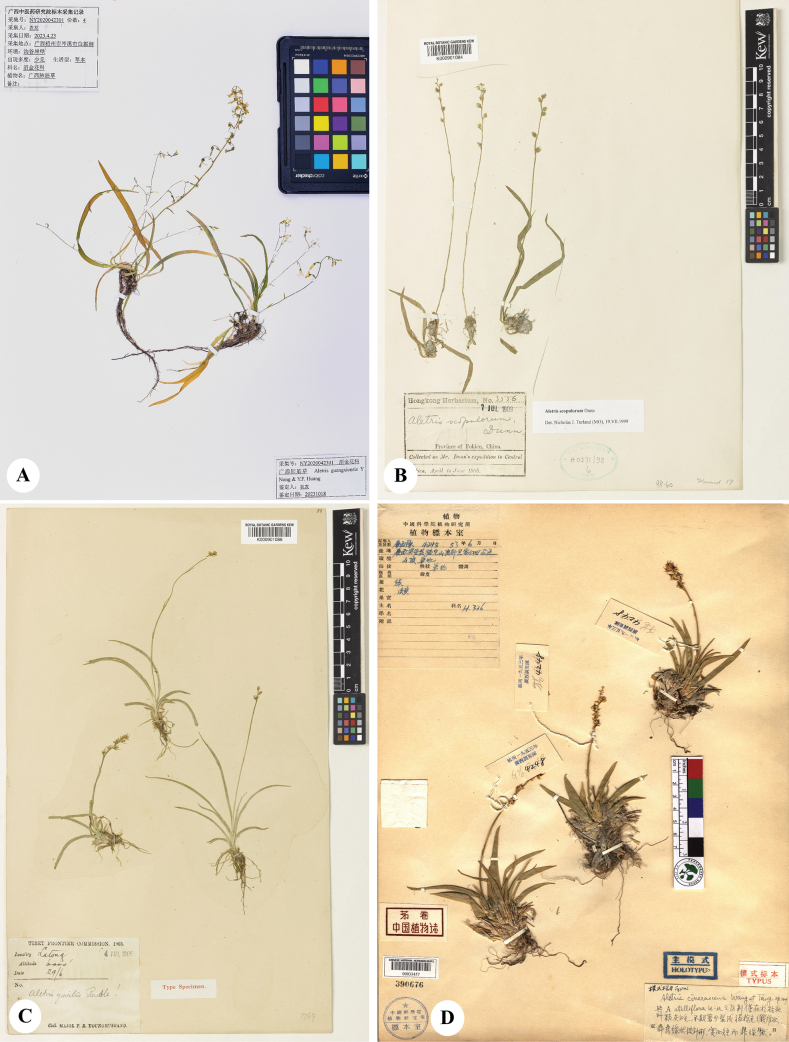
Digital images of type specimens **A***Aletrisguangxiensis* [*Y Nong NY2020042301* (GXMI!)] **B***A.scopulorum* [*Dunn 3556* (K!)] **C***A.gracilis* [*Younghusband s.n.* (K!)] **D***A.cinerascens* [*Guangxi Investigation Team 4248* (PE!)].

##### Distribution and habit.

**Figure 5.**
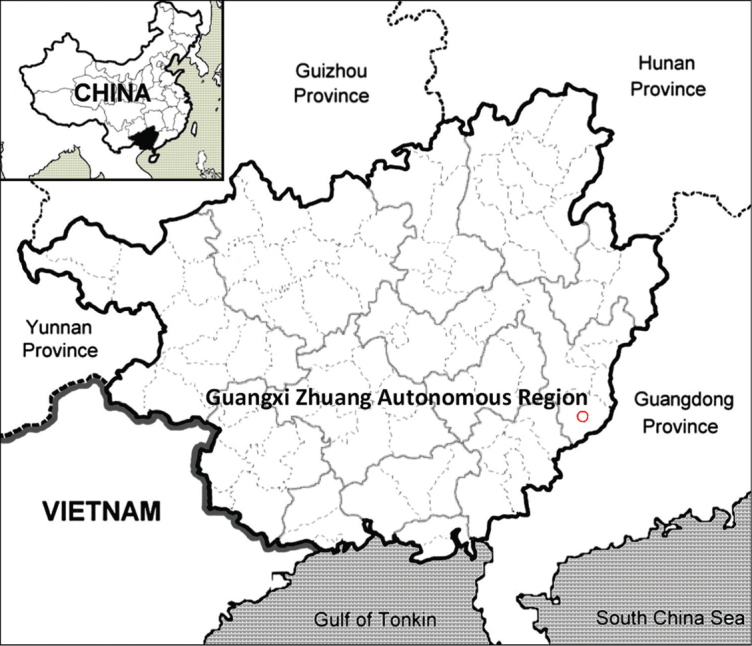
The distribution of *Aletrisguangxiensis* (red circle) in Guangxi, China.

Known only from the southeast of Guangxi, China (Fig. 5). The new species mainly occurs at elevations of 320 m. It has been mainly found on moist cliffs next to streams.

##### IUCN Red List Category.

Data available for the new species are still insufficient to assess its conservation status. According to the IUCN Criteria (IUCN 2022), it is considered Data Deficient (DD) until more information becomes available. Although the population of *A.guangxiensis* is currently in relatively good conditions, further collection and monitoring are necessary to allow more conclusive estimations about the rarity and vulnerability of the species. Therefore, special attention should be given to the conservation of the new species of *Aletris*.

##### Additional specimen.

Cenxi. Southeast Guangxi: limestone hills, fl. 30 March 2023, *G.Y. Wei WGY2023033001* (GXMI!).

### ﻿Key to species of *Aletris*

**Table:** 

1	Flowers usually solitary, rarely densely 2–or 3–flowered forming a raceme	**1. *A.simpliciflora***
–	Flowers usually densely 4–14–flowered forming a raceme	**2**
2	Perianth abaxial surfaces rough	**3**
–	Perianth glabrous or pubescent	**8**
3	Perianth usually wholly yellow to golden yellow, rarely white	**4**
–	Perianth white to creamy–white, lobes sometimes tipped with orange or pinkish–orange	**6**
4	Perianth campanulate, 6–7 mm, 2 times or less as long as broad	**2. *A.aurea***
–	Perianth cylindrical, 9–12 mm, more than 2.5 times as long as broad	**5**
5	Lobes spreading	**3. *A.lutea***
–	Lobes erect	**4. *A.×tottenii* (*A.lutea ×A.obovata*)**
6	Perianth campanulate or obovoid, lobes turned slightly inwards	**5. *A.obovata***
–	Perianth cylindrical, lobes spreading	**7**
7	Leaves dull greyish–green, 0.6–1 cm wide; beaks of fruits gradually tapering from body to tip	**6. *A.bracteata***
–	Leaves bright yellowish–green, 0.5–2.6 cm wide; beaks of fruits abruptly narrowed distally	**7. *A.farinosa***
8	Perianth pubescent, sometimes sparsely or minutely so	**9**
–	Perianth glabrous, rarely papillose	**15**
9	Leaves 1–1.5 cm wide; perianth 7–10 mm	**8. *A.megalantha***
–	Leaves less than 1 cm wide; perianth less than 7 mm	**10**
10	Bracts 2–5 × flower length	**9. *A.glandulifera***
–	Bracts shorter than or subequalling flower length, sometimes a few bracts near base of raceme to 2 × flower length	**11**
11	Flowers usually subsessile, pedicels absent to 1(–2) mm, bract and bracteole borne on distal 1/2 of pedicel (often near apex); perianth lobes linear–lanceolate or narrowly oblong–lanceolate to linear; capsule turbinate, oblong–obovoid, obovoid or ovoid	**12**
–	Flowers distinctly pedicellate, pedicels 0.5–3.5 mm, bract and bracteole borne on proximal 1/2 of pedicel (often near base); perianth lobes ovate to lanceolate; capsule subglobose	**13**
12	Capsule turbinate, oblong–obovoid or obovoid, distinctly angular, 3–5 × 2–3 mm, abruptly contracted distally when dehisced; leaves 2–4(–5) mm wide	**10. *A.spicata***
–	Capsule ovoid, not angular, 4–6 × 3–4.5 mm, not or only slightly contracted distally when dehisced; leaves (2–)3–5(–8) mm wide	**11. *A.stenoloba***
13	Leaves 1–5, laxly tufted; rhizome cormlike, 3–7 mm in diam	**12. *A.scopulorum***
–	Leaves numerous, densely tufted; rhizome not corm–like	**14**
14	Perianth lobes oblong–lanceolate, 2–3 mm	**13. *A.pedicellata***
–	Perianth lobes ovate, ca. 1 mm	**14. *A.yaanica***
15	Raceme axis and pedicels glabrous	**16**
–	Raceme axis and pedicels pubescent or puberulent	**20**
16	Raceme covered with viscid secretion;perianth tube urceolate, strongly constricted at apex, lobes erect	**17**
–	Raceme not covered with viscid secretion; perianth tube broadly funnelform, lobes strongly recurved or revolute	**18**
17	Pedicel 0.5–3(–4.5) mm; bract 2–16 mm, perianth yellowish–green or cream 3–6 mm	**15. *A.glabra***
–	Pedicels 1 mm; bracts 5–15 mm long, yellow green corollas 6–7 mm long	**16. *A.foliata***
18	Rhizome surrounded by mass of fibres from disintegrated leaf bases; capsule with persistent stigma conspicuously thickened and capitate	**17. *A.gracilis***
–	Rhizome not surrounded by mass of fibres, but sometimes by persistent, dead leaves; capsule with persistent stigma not or only slightly thickened	**19**
19	Capsule oblong–ovoid or ± ellipsoid, 5–7 × 3–3.5 mm	**18. *A.cinerascens***
–	Capsule ellipsoid or ovoid, to 7 mm long	**19. *A.foliolosa***
20	Bracteole borne on proximal 1/2 of pedicel (often near base)	**20. *A.guangxiensis***
–	Bracteole usually borne on distal 1/2 of pedicel (often near apex)	**21**
21	Rhizome often surrounded by mass of fibres from disintegrated leaf bases; roots thickened, fleshy; leaves usually rather few (5–10) and laxly tufted; capsule ovoid ellipsoid or ovoid–conical	**21. *A.pauciflora***
–	Rhizome not surrounded by mass of fibres; roots fibrous; leaves numerous and densely tufted; capsule narrowly ovoid to subglobose	**22**
22	Raceme densely capitate or oblong–capitate; bract and bracteole borne on proximal 1/2 of pedicel (often near base)	**22. *A.capitata***
–	Raceme elongate and lax to short and dense, but not capitate; bract and bracteole usually borne at or near apex of pedicel	**23**
23	Perianth 4–7.5 mm, lobes 2–5.5 mm, erect, spreading, recurved or revolute, 1–5× tube length	**23. *A.laxiflora***
–	Perianth 3–4.5 mm, lobes 1–2 mm, erect or recurved, 0.3–1× tube length	**24**
24	Scape very slender, wiry, often somewhat flexuous, 7–20 cm; bract shorter than perianth; perianth often densely papillose, lobes recurved	**24. *A.alpestris***
–	Scape relatively stout, not wiry, straight and erect, 1.5–10 cm; bract equalling or longer than perianth; perianth not or scarcely papillose, lobes erect or slightly recurved	**25. *A.nana***

## Supplementary Material

XML Treatment for Aletris guangxiensis

## ﻿Additional information

### Author ORCIDs

You Nong https://orcid.org/0000-0001-7004-0946

Yun-Rui Qin https://orcid.org/0000-0003-2692-9048

Gui-Yuan Wei https://orcid.org/0000-0003-0652-1213

Ke-Jian Yan https://orcid.org/0000-0002-4927-4665

Chuan-Gui Xu https://orcid.org/0009-0000-6263-3821

Zi-Yi Zhao https://orcid.org/0000-0003-2513-0728

Ren-Chuan Hu https://orcid.org/0000-0002-0941-7203

### Data availability

All of the data that support the findings of this study are available in the main text.
